# The Effect of Noises upon Certain Forms of Deafness

**Published:** 1884-08

**Authors:** George Hawley

**Affiliations:** Chicago, late House Surgeon at Golden Square Throat and Chest Hospital, London, England, Assistant Surgeon to the Illinois Eye and Ear Infirmary


THE CHICAGO

MEDICAL JOURNAL AND EXAMINER.

VOL. XLIX. AUGUST, 1884. NO. 2.

### ORIGINAL COMMUNICATIONS.

ARTICLE I.

### THE EFFECT OF NOISES UPON CERTAIN FORMS OF DEAFNESS.

*By* GEORGE HAWLEY, M. D., *Chicago, late House Surgeon at Golden Square Throat and Chest Hospital, London, England, Assistant Surgeon to the Illinois Eye and Ear Infirmary.*

[*Read before the Chicago Medical Society, June*, 1884.]

More than two hundred years ago Dr. Thomas Willis, of Amsterdam, described a certain form of deafness in which hearing was improved by noise enabling persons to distinguish tones and sentences which at other times would be unheard. This symptom, bearing the name of Paracussis Willisiana, has been accepted by most aurists, but no satisfactory explanation as to its cause has been advanced.

While Politzer, von Troeltsch and others consider that it depends upon some change in the position of the ossicles or membrana tympani, many, like Krama, take different views and believe that it is due to a stimulating of the auditory nerve, thus forcing it to a more healthy action. Experience favors the theory that the middle ear is the seat of this symptom, that some change is brought about by the constant noise which restores the hearing for the time being. What this change is, is still open to discussion. It may be of interest to relate the following case which came under my observation while house surgeon at the Golden Square Hospital, London. Mrs. H., aged 30, had for the last five years suffered with marked deafness in both ears. Common conversation was heard with difficulty except when any loud noise was present. At that time the hearing apparently was greatly improved. A watch could be heard on the left side only upon contact, and on the right at a distance of about two inches. The tuning-fork showed no trouble of the acoustic nerve, but clearly indicated some disturbance in the middle ear. The drum-head was normal as to tension, color, etc., while perfect movability existed in the malleo-incudal joint. Nothing indicated anchylosis of of any of the ossicles. The appearance of the throat was healthy and the eustachian tubes both patent. This is a good example of this form of deafness in which the symptom of hearing better in a noise manifests itself.

Roosa, however, mentions two cases where the drum-head was more or less destroyed and still, noises improved the hearing. The ossicles in both ears were intact. Politzer, in his work on aural surgery, states that this improvement is seen only in middle ear affections of an adhesive or sclerotic character, and regards it as a most unfavorable symptom for satisfactory prognosis. Roosa, on the contrary, states that it is found in many forms of ear affections, and does not necessarily forbid one from expecting favorable results from treatment. In his article on "The Effects of Noises upon Diseased and Healthy Ears," he says, "I have known two cases where this symptom occurred in patients who regained their hearing perfectly. While the symptom frequently accompanies incurable diseases of the middle ear, I believe it is a very frequent symptom in sub-acute cases where both ears are affected. Of course it would not be observed in disease of one ear only."

Whatever may be the cause of this symptom, we are justified, I think, in believing that certain pathological changes are then present in the middle ear, which will be found at no other time. If this be true, in what does the change consist?

Willis considered the change brought about by the action of strong wave tones upon a relaxed membrana tympani to be the cause of this symptom. As long as the drum-head is in a relaxed condition it refuses to respond fully to wave impressions. It is no longer a perfect sound-conductor until its tension is restored. Noises, by forcing air against the membrane, press it inward, restore its tension, and hearing is improved until the pressure is removed, when deafness returns as bad as ever. If this improvement was found only in such cases as he describes, we might accept his theory as correct; but, unfortunately, as I have already stated, it is present with no change whatsoever in the drum-head. Therefore, in such cases, at least, this improvement in hearing is due to some other cause. We have in the middle ear two other elastic membranes, one of which takes as important a part in conveying wave impressions as the membrana tympani. Any change by disease or injury would be fully as disastrous to normal hearing; I mean the membrane of the foramen ovale.

When we consider the extraordinary fine shades of vibrations transmitted by this membrane to the fluids of the labyrinth, are we not justified in expecting that any loss of accuracy and precision on the part of the transmitting apparatus at this point will cause a corresponding loss of hearing power. According to Riemann the excursions of the stapes in the fainter tones are so small as to escape detection even with the highest power of the microscope. Therefore a corresponding sensitiveness on the part of the membrane must necessarily be present in order to appreciate and transmit these vibrations. Thus we see that any change at the oval window which tends to hinder the passage of vibrations to the fluids in the inner ear must cause diminution in hearing. If, then, the membrane from any cause becomes relaxed, and loses the power of responding to vibrations, it acts no longer as a sound conductor but as a damper to any wave impressions.

This is demonstrated in the case of relaxed drum-heads where inflation improves the hearing. Not only must this elastic plate (so called by Politzer) be capable of responding fully to any wave impulse, but it must also bear a certain relation to the foot of the stapes. I mean by this that a due amount of pressure must be applied by the stirrup and a sufficient resistance be offered in every ear that possesses perfect hearing. If this is lost, deafness is the result. Chronic suppurative inflammation of the middle ear, with destruction of drum-head, incus and malleus, is a proof of the correctness of this statement. Hearing is here frequently improved by a false drum-head, which presses the loose stapes against the membrane of the foramen ovale, thereby restoring the relative positions of the parts. In a relaxed membrane the pressure of the stapes is wanting, due to an absence of resistance in the diseased membrane itself.

Until we find some means of restoring this needed resistance, deafness remains. Thus through the loss of elasticity in this membrane we lose two important factors for perfect hearing. First, this membrane, like the membrana tympani, while relaxed, refuses to act as a conductor of sound by not responding to vibrations given it. And second, the proper relation between it and the stapes is destroyed, the necessary pressure and resistance being absent. We find in the case of an atrophied drum-head that hearing is frequently improved by inflating the middle ear, which restores the tension to this membrane by forcing it outwards. One might suppose that such inflation would likewise restore lost hearing if due to a relaxed membrane at the ovale window. Unfortunately, the same act which might restore to this membrane its proper tension, forces it inwards as well, and thus separates the stapes from it.

Though the membrane may be in a proper condition for receiving and transmitting wave sounds, the stapes, from the absence of any pressure applied by it to this membrane, will be unable to perform its duty—there will be more or less obstruction to the passage of sound at this point.

Whatever, then, restores the hearing in these cases. must not only renew the tension of the membrane, but maintain as well the relative position of health between it and the stapes.

This, in my opinion, can only be accomplished by some influence brought to bear upon the membrane and stapes from outside the middle ear.

If now we consider the effect of wave impulses upon the membranes and ossicles, we can, I think, understand how a loud and continuous noise might improve the hearing where deafness is due to such pathological changes as herein described.

The air, set in motion by this noise, strikes upon and pushes the drum-head and ossicles inwards; this crowds the air in the middle ear against the ovale window, and the membrane, if relaxed, is made tense by this pressure. At the same time, the relative position between it and the stapes, as to pressure, etc., is also restored, and hearing for the time is therefore improved.

This noise, which would confuse one with normal hearing, is absorbed, so to speak, in removing the cause of deafness.

In case of perforated drum-head the vibrating air acts directly upon the diseased membrane and stapes.

Von Troeltsch mentions a case where a magistrate so afflicted improved his hearing by pressing a bit of stick against the drum-head. Allen, also, in his work on Aural Surgery, states, while speaking of this symptom, that the hearing power was increased in a patient who presented herself with marked destruction of the drum-head by pressing cotton against the ossicles. Another is also noted where a large perforation existed. Here, before and after healing of the perforation, hearing was improved by pressure.

Though these three cases do not in themselves place this theory beyond dispute, it may still make it worthy of consideration.

It may encourage us to further investigations, which, perchance, may result in establishing beyond a doubt the cause of improved hearing in certain forms of deafness, by noise.

